# Current-induced magnetic switching with spin-orbit torque in an interlayer-coupled junction with a Ta spacer layer

**DOI:** 10.1038/s41598-018-22122-1

**Published:** 2018-02-28

**Authors:** W.-Y. Kwak, J.-H. Kwon, P. Grünberg, S. H. Han, B. K. Cho

**Affiliations:** 1 School of Materials Science and Engineering, Gwangju Institute of Science and Technology (GIST), Gwangju, 61005 Republic of Korea; 2 Grünberg Center for Magnetic Nanomaterials, Gwangju Institute of Science and Technology (GIST), Gwangju, 61005 Republic of Korea; 3 Division of Navigation Science, Mokpo National Maritime University, Mokpo, 58628 Republic of Korea

## Abstract

Spin-orbit torque has attracted considerable attention as a means to overcome limits of devices based on spin-transfer torque. However, a small magnetic field that is collinear to the current flow must be applied to break symmetry and induce deterministic current-induced magnetization switching. Recently, a junction utilizing interlayer coupling mediated by a Ru spacer layer between two CoFe layers was designed for symmetry breaking and exhibited current-induced magnetization switching without a magnetic field. Here, we demonstrate zero-field current-induced switching of the perpendicular magnetization of a Co layer that is indirectly coupled with a CoFe layer via a Ta spacer. The weak interlayer coupling exhibited by Ta allows the layer thickness to be relatively small (≈0.5 nm), enabling appropriate interlayer coupling to induce spin-orbit torque for current-induced magnetic switching. External magnetic field effects on switching characteristics show that the current switching process is quite stable against external environments.

## Introduction

The manipulation and detection of magnetization in ferromagnetic layers using spin-transfer torque (STT) have received considerable attention for their potential applications in spintronic devices^1–6^. A new phenomenon known as spin-orbit torque (SOT) was recently designed to switch magnetization using an electric current, similar to the effect of STT, for heavy metal/ferromagnet bilayers^7–12^. The spin Hall effect (SHE), which is responsible for SOT, generates a transverse spin current that is induced by the numerical imbalance of deflected spin-up and spin-down electrons in a heavy metal owing to strong spin-orbit coupling^13^. The spin current is absorbed by an adjacent ferromagnetic layer, resulting in both a Slonczewski-like torque (SLT) and a field-like torque (FLT)^10,14^. Neither torque possesses an up- or down-directional preference, but if a magnetic field is applied collinearly to the current, the effective field from SLT is directed perpendicular to the junction plane wherein electric current flows^15,16^. In consideration of its applications, SOT switching has advantages in terms of the writing current, energy efficiency, and scalability compared with those of conventional STT switching^5^.

However, the collinear magnetic field should be eliminated because of its detrimental effects on neighbouring circuits, which become more serious as a spin-device moves to higher density. Thus far, a few magnetic junction techniques have been designed to achieve zero-field SOT switching: breaking lateral structural symmetry using the thickness gradient along the direction transverse to the current flow within the plane^17^, tilting an easy axis from its initial perpendicular direction using a wedge-shaped ferromagnetic layer^18^, controlling domain wall motion in a ferromagnetic layer using a distinctive shape^19^, tilting magnetization using direct exchange coupling with an antiferromagnet^20–22^, and interlayer coupling using an in-plane magnetized ferromagnet^23^.

Of these techniques, the interlayer coupling structure exhibits the most robust deterministic SOT switching against external conditions. In this structure, the thickness and material of a spacer layer are important parameters affecting the switching performance of a device because interlayer coupling determines the characteristics of SOT and the interlayer exchange coupling strength changes exponentially depending on the number of d-electrons in a transition metal layer within ferromagnet/transition metal multi-layers^24^. Recently, Ru was used in a spacer layer to show robust zero-field switching of a Co layer with perpendicular magnetic anisotropy. It seems plausible to utilize Ru, which has an electronic configuration of 4d^7^5s^1^, because it is an element that induces strong interlayer exchange coupling. However, strong interlayer coupling destroys the perpendicular magnetic anisotropy of the under-layer; thus, the thickness of the Ru layer should be optimized to maintain a minimum value of 2.5 nm^23^. Therefore, it would be interesting to replace the Ru spacer layer with an element, e.g., Ta, typified by a weaker interlayer coupling strength, to investigate the characteristics of SOT switching. The element Ta, which has an electronic configuration of 5d^3^6s^2^, would induce the weakest possible exchange coupling strength within Co/transition metal multi-layers among 3d, 4d and 5d transition metals, and therefore, the thickness of a Ta spacer layer can be minimized to maintain the appropriate interlayer coupling. In addition, considering industrial demand, it is worth to mention that Ta is much cheaper than Ru.

Here, we investigated the characteristics of SOT switching within a stack of layers consisting of Ta/Pt/Co/Pt/Ta/CoFe/IrMn/Ta, wherein the middle Ta layer is a spacer layer for interlayer coupling between Co and CoFe. Ta was found to induce ferromagnetic coupling for almost every thickness range. Thus, the junctions in this study were fabricated with the Ta spacer layer thickness of 0.5 nm, showing ferromagnetic coupling between ferromagnetic layers. The thin Ta spacer layer shows optimum interlayer coupling to induce stable magnetic switching with current only.

## Results and Discussion

### Fabrication and magnetic properties of the sample

**Figure 1:**
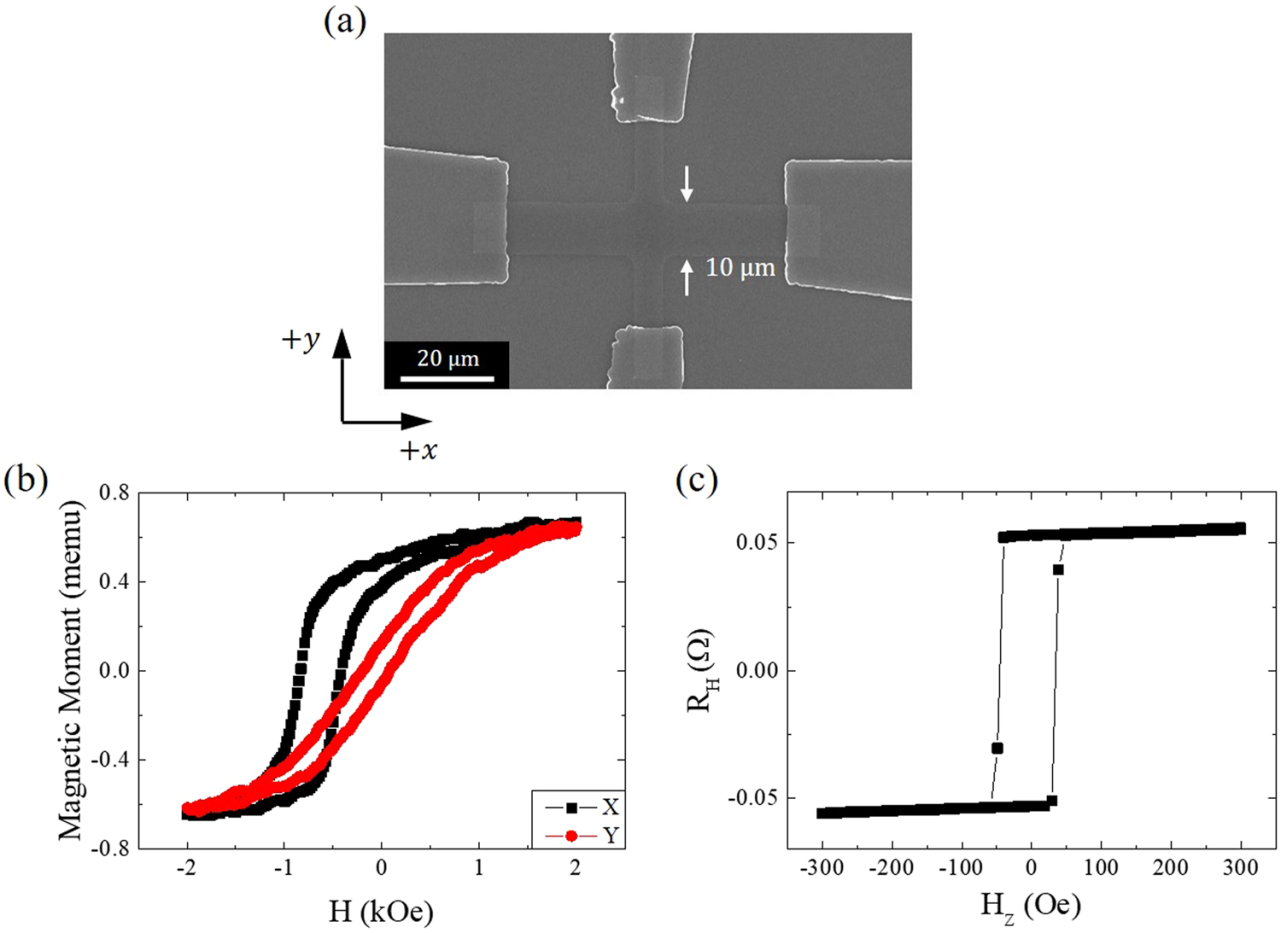
Fabrication and magnetic properties of the sample. (**a**) Scanning electron microscopy image of the Hall bar. Electrical measurements were conducted with current injection along the *x*-axis and with voltage detection along the *y*-axis through the contact line over the Hall bar. See the text for a description of the junction structure. (**b**) Isothermal magnetization hysteresis curves of a film, which were obtained by vibrating a sample magnetometer along the *x*-axis (square) and *y*-axis (circle) when the CoFe layer was pinned along the *x*-axis. The film is patterned in the form of a Hall bar whose longitudinal axis is the same as the pinned CoFe axis. (**c**) Anomalous Hall effect data of the Hall bar. A constant DC current of 1 mA was applied along the *x*-axis to detect the Hall voltage, which corresponds to the magnetization reversal of Co with the applied magnetic field perpendicular to the *xy* junction plane.

Figure 1(a) shows a scanning electron microscopy (SEM) image of a Hall bar with a magnetic multi-layer consisting of the following: Ta(3 nm)/Pt(5 nm)/Co(0.6 nm)/Pt(0.4 nm)/Ta(0.5 nm)/CoFe(3 nm)/IrMn(15 nm)/Ta(1 nm). The Hall bar, with a width of 10 μm and a length of 60 μm, is fabricated using electron-beam lithography and an ion-milling process and is deposited with a base pressure of 8 × 10^−9^ Torr using a DC magnetron sputtering system. The thickness of each layer is controlled by the deposition time based on the growth rate, which is determined using X-ray reflectivity measurements. The bottom Ta layer is used as a buffer layer, and the top Ta layer protects the structure from oxidation. The Co exhibits PMA because of two adjacent interfaces with Pt layers. The upper Pt layer has a minimum thickness allowing PMA in the Co layer, and the lower Pt layer plays a role as a spin current source in addition to PMA. The spin currents, which are generated from the two Pt layers, are not cancelled out because of their different thicknesses^25–27^. In this system, a thickness of 0.5 nm for the Ta spacer layer was found to be the minimum thickness to establish a strong interlayer interaction because Co loses its PMA in a structure characterized by a thickness of less than 0.5 nm for the Ta layer (see the Supplementary Information for details). Figure 1(b) illustrates the isothermal magnetization curves of a CoFe layer when a magnetic field is applied along the *x*- and *y*-axes, where the *x*-axis is parallel to the pinned magnetization of CoFe. An exchange bias of ≈620 Oe at the interface between the CoFe and IrMn layers is defined from the hysteresis shift in the *x*-axis isothermal curve. The relatively linear magnetization loop along the *y*-axis is consistent with the fact that the magnetization of the CoFe layer is pinned along the *x*-axis. A magnetization reversal of the Co layer exhibiting PMA is electrically detected by an anomalous Hall effect (AHE) measurement^28^, which produces a voltage difference depending on the magnetization alignment (either up or down) as a function of the magnetic field perpendicular to the Hall bar plane. To acquire a Hall measurement, we applied a constant DC current along the *x*-axis and detected the voltage difference *V*_*H*_ along the *y*-axis. An anomalous Hall data and hysteresis loop, as illustrated in Fig. 1(c), can be understood in terms of Co magnetization switching through an applied field. A coercive field of approximately 40 Oe of Co is estimated from the hysteresis Hall curve.

### Current polarity effects on switching behaviour

**Figure 2:**
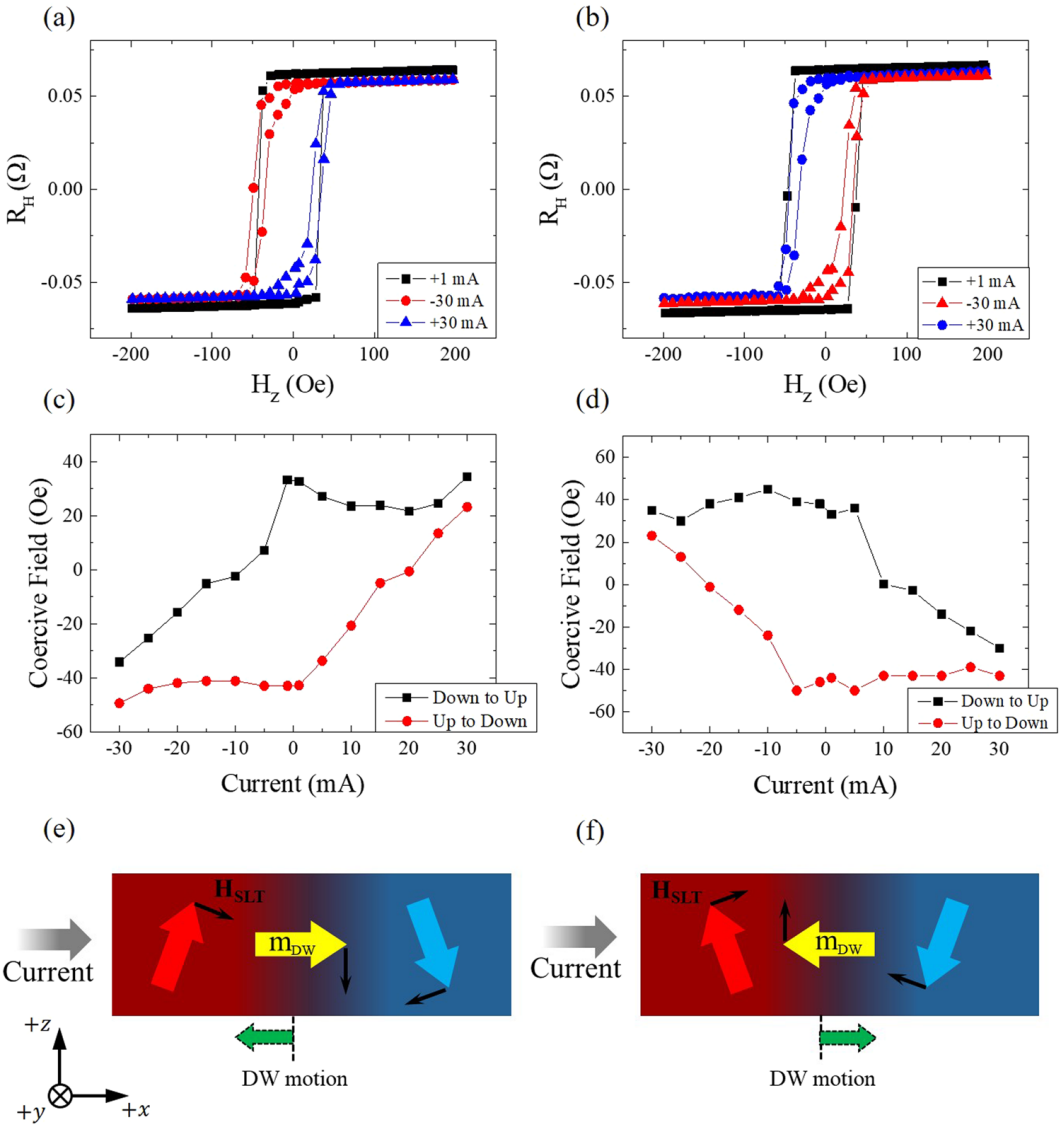
Current polarity and amplitude effects on switching behaviour. (**a**,**b**) Anomalous Hall effect resistance in terms of the applied magnetic field along the *z*-axis when DC currents of 1 mA and ±30 mA flow along the *x*-axis in a multi-layer junction (see text) with the CoFe layer pinned along the *x*-axis and –*x*-axis, respectively. (**c**,**d**) Coercive field for switching of the Co layer with respect to the injected current amplitude in the junction structures of (a) and (b), respectively. (**e**,**f**) Schematic spin configurations of the domain and domain wall of the Co layer (large arrows), domain wall motion (dotted arrows), and the effective field due to spin-orbit torque (small arrows) when current is flowing along the *x*-axis in the junction structures of a and b, respectively.

Figure 2 shows in-plane current effects on the AHE data and the coercive field of Co as well as a schematic configuration of electric and magnetic parameters. We applied DC currents of ±1, ±5, ±10, ±15, ±20, ±25, and ±30 mA along the *x*-axis while simultaneously sweeping the perpendicular magnetic field. For representative data, Fig. 2(a) shows AHE hysteresis curves when CoFe is pinned along the *x*-axis under applied currents of 1 mA and ±30 mA. The resulting hysteresis indicates that the coercive fields of Co have values of ≈35 Oe and ≈−50 Oe when the magnetic reversal occurs from down to up and from up to down, respectively, assuming no significant effect of the 1 mA current. When a current of – 30 mA is applied, the coercive field for the magnetization reversal from down to up changes from ≈35 Oe to ≈−33 Oe, whereas there is almost no change in the reversal field (≈−50 Oe) from up to down. When a current of 30 mA is applied, the magnetic reversal from up to down occurs at 22 Oe, rather than ≈−50 Oe, and there is no change in the reversal field from down to up at 35 Oe. Figure 2(b) shows the same data as Fig. 2(a) when CoFe is pinned along the −*x*-axis. A polarity change of CoFe induces the opposite current effect on the magnetic reversal of Co. The positive current induces a shift of the reversal field from down to up, and a negative current induces a shift of the reversal field from up to down. This behaviour indicates that the current that flows in the Pt layer exerts a significant influence on Co magnetization combined with the pinned magnetization of CoFe. Figure 2(c,d) plot the coercive fields for a current with CoFe pinned along the positive and negative *x*-axis, respectively. A positive current with CoFe pinned along the *x*-axis has almost no effect on the coercive field for magnetic reversal from down to up, whereas the coercive field from up to down shows a nearly linear dependence on the positive current. The negative current induces a similar coercive field change, but it is opposite to the positive current. When the CoFe layer is pinned along the negative *x*-axis, the current dependence of the coercive field is opposite to that when CoFe is pinned along the positive *x*-axis.

This phenomenon can be explained by the existence of spin-orbit torque^17,20^, which is induced by the combination of spin current in the Pt layer and interlayer coupling between the Co and CoFe layers^23^. Interlayer coupling between the Co and CoFe layers tilts the magnetization of the Co layer along the same direction as the pinned CoFe layer and also stabilizes the Néel wall rather than the Bloch wall, which is energetically favourable in a perpendicularly magnetized ferromagnetic layer. Torque, which is generated by the absorption of spin current at the heavy metal/ferromagnet interface and acts upon local magnetization, is either SLT or FLT. The two orthogonal torques vary with both the thickness and the material composition of each layer comprising the device^8,10–12,25,27^. The effective field related to FLT has $(\hat{{z}}\times \hat{{{j}}_{{e}}})$ symmetry, where $(\hat{{z}}\times \hat{{{j}}_{{e}}})$ are unit vectors in the direction perpendicular to the plane and electron flow, respectively, meaning that the ef fective field has a fixed direction regardless of the magnetization in the domain wall for a particular current flow^10,26,27^. The effective field corresponding to SLT is given by ${\mathop{H}\limits^{\rightharpoonup }}_{SLT}={H}_{SLT}^{0}(\hat{{m}}\times (\hat{{z}}\times \hat{{{j}}_{{e}}}))$, where $\hat{m}$ is the unit vector of magnetization in the domain wall. The magnitude of the effective field is expressed as ${H}_{SLT}^{0}=\hslash {\theta }_{SH}|{j}_{e}|$/(2 $|e|{M}_{S}{t}_{F}$), where ${\theta }_{SH}$ is the spin Hall angle of the heavy metal, and ${M}_{S}$ and ${t}_{F}$ are the saturation magnetization and thickness of the ferromagnetic layer, respectively. With SLT symmetry, the current flowing along the *x*-axis creates a counter-clockwise (clockwise) effective field about the *y*-axis because of a positive spin Hall angle for Pt within the structure with CoFe pinned along the *x*-axis (−*x*-axis), resulting in a decreased coercive field from up to down (down to up). Therefore, the effects of current and of pinned CoFe magnetization polarities on the switching of Co, as illustrated in Fig. 2, can be understood with respect to SLT, and the current dependence of the coercive field is also explained.

### Deterministic SOT switching without external magnetic field

**Figure 3:**
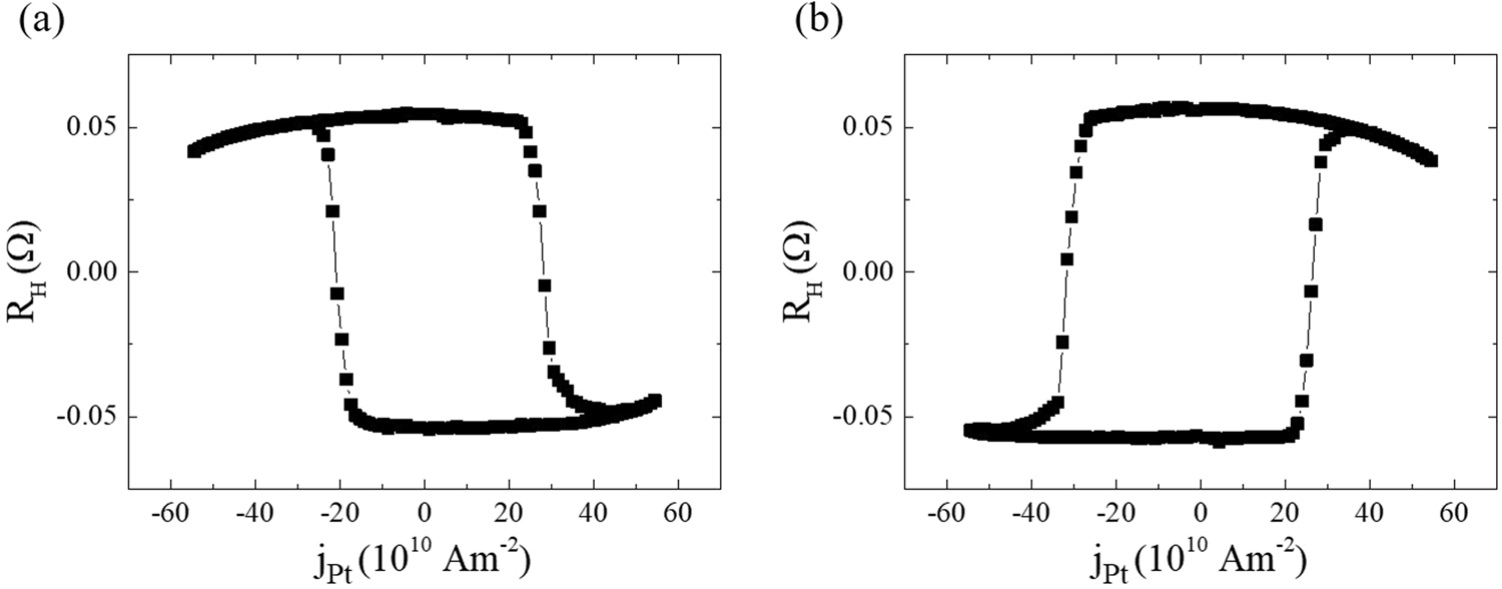
Deterministic spin-orbit torque (SOT) switching without an external magnetic field. (**a**,**b**) Anomalous Hall resistance as a function of pulsed current density of 1 ms duration along the *x*-axis in a multi-layer junction (see text) with the CoFe layer pinned along the *x*-axis and –*x*-axis, respectively.

Next, we study current-induced magnetization switching without an external magnetic field. For current-induced switching and related measurement acquisition, a pulsed current with 1 ms duration is successively injected from −40 mA to +40 mA with a step of 0.8 mA. At the centre of an injected pulse current, the Hall voltage difference is measured to detect the magnetization state (i.e., whether it is up or down) in the Co layer. Figure 3(a,b) illustrate the deterministic switching of Co without an external magnetic field, wherein CoFe is pinned along the positive and negative *x*-axis, respectively. As described above, the nucleated domain wall has an internal magnetization along the CoFe pinned direction owing to interlayer coupling between the Co and CoFe layers, resulting in the configuration of up-right-down-right-up or up-left-down-left-up when the CoFe is pinned along the +*x*-direction and −*x*-direction, respectively. Since the fast and steady motion of the Néel wall resulting from spin torque, which is induced by SHE within a heavy metal, has been theoretically demonstrated for a strip composed of heavy metal/ferromagnet bilayers, stabilization of the Néel wall is an essential prerequisite in SOT switching. According to SLT symmetry, the domain wall aligned along the *x*-axis generates an effective field along the *z*-axis (−*z*-axis) when pulsed current is injected along the −*x*-axis (*x*-axis). By contrast, an effective field along the *z*-axis (−*z*-axis) is produced when pulsed current is applied along the *x*-axis (−*x*-axis) when the domain wall is aligned along the −*x*-axis. The perpendicular effective field induces the expansion of domains where magnetization is aligned in the same direction as that of the effective field, whereas domains with opposite magnetization to the perpendicular effective field shrink, rather than domain wall displacement. Thus, non-chirality of spins in domain walls due to the interlayer coupling leads to a complete magnetization switching of the Co layer^15^. Consequently, clockwise and anticlockwise loops of the Hall voltage (with respect to the current) are obtained when CoFe is pinned along the *x*-axis or −*x*-axis, respectively, as shown in Fig. 3. The corresponding magnetization switching is consistent with the switching result when Pt produces a spin current and an external magnetic field is applied along the *x*-axis and −*x*-axis^9,26^.

### Current-induced magnetization switching under an external magnetic field along the ±*x*-axis

**Figure 4:**
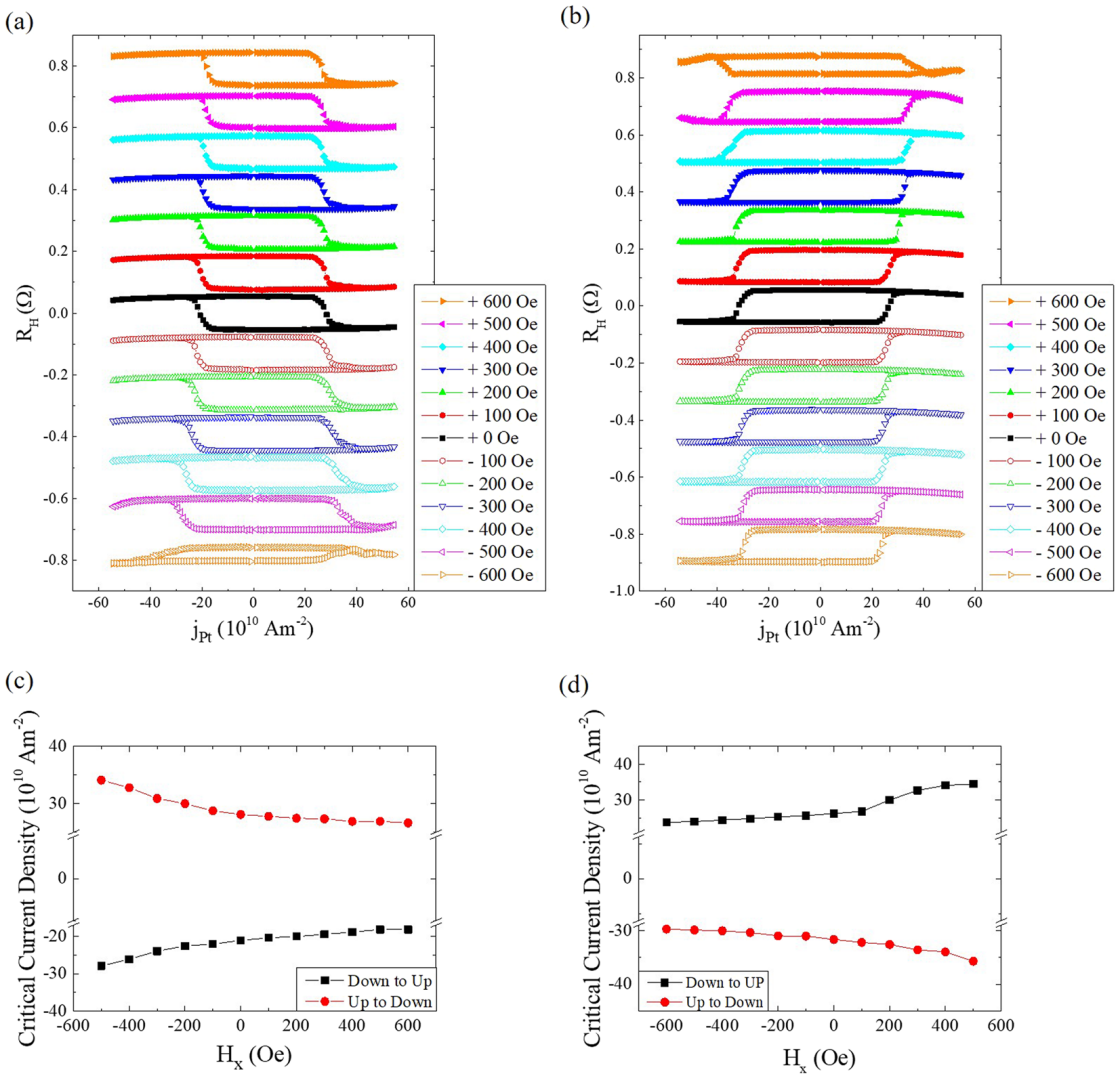
Current-induced magnetization switching under an applied magnetic field along the ±*x*-axis. (**a**,**b**) Anomalous Hall resistance in terms of pulsed current density of 1 ms duration coincident with the application of an external field, −600 Oe ≤ *H* ≤ +600 Oe, in multi-layer junctions (see text) with the CoFe layer pinned along the *x*-axis and –*x*-axis, respectively. (**c**,**d**) Variation of critical current density for Co layer switching in terms of the magnetic fields in (**a**) and (**b**), respectively.

To examine the stability of the junction possessing an ultrathin Ta spacer, a constant magnetic field within the range of −600 Oe ≤ *H* ≤ +600 Oe was applied along the *x*-axis together with a pulsed current injection. The pulsed current with a duration of 1 ms was injected along the *x*-axis across a range from −40 mA ≤* I* ≤ +40 mA with a step of 0.8 mA. Figure 4(a) displays the Hall resistance in terms of the pulsed current, i.e., current-induced magnetization switching, for the junction with CoFe pinned along the *x*-axis. Each Hall resistance-current loop was obtained under constant magnetic fields over the range −600 Oe ≤ *H* ≤ +600 Oe with a step of 100 Oe. With the application of a magnetic field along the *x*-axis, there is no observed change in current switching behaviour, i.e., the Hall resistance-current loops under an applied field are almost identical to those under a zero field (regardless of the magnitude of the magnetic field). By contrast, the switching currents are observed to increase with an increase of the applied magnetic field along the −*x*-axis, while the switching process keeps clockwise for *H* ≥ −500 Oe. For *H* = −600 Oe, the switching process changes from clockwise to anticlockwise, which is consistent with the fact that the CoFe layer is pinned along the *x*-axis by an exchange interaction with IrMn characterized by a bias field of ≈600 Oe, as shown in Fig. 1(b). This change in the switching sequence indicates that the magnetic polarity of the CoFe layer is reversed by the applied field from the positive to the negative *x*-axis. The hysteresis with *H* = −600 Oe exhibits an incomplete magnetization reversal of the Co layer, leading to a lower *R*_*H*_ value than those with other applied magnetic fields. Figure 4(c) plots the critical current density for Co magnetization switching as a function of the axial magnetic field. The critical current density is defined as the current density value at the halfway point during the switching process. The critical current density decreases (increases) with an increase in the positive (negative) applied fields. As shown in Fig. 4(c), the quantity of critical current density decreases with the positive field is relatively small (2~3 $\times {10}^{10}{{\rm{Am}}}^{-2}$), compared to that of the critical current density increase observed with a negative field (5~6 $\times {10}^{10}{{\rm{Am}}}^{-2}$), which indicates that the field that is antiparallel to the exchange coupling has a greater effect on the current-induced switching than the parallel field. This change in the critical current density depending on the direction of the applied magnetic field can be understood in terms of the interlayer coupling between the Co and CoFe layers. Within the applied field parallel (antiparallel) to interlayer coupling, the critical current decreases (increases) because a higher magnetic field leads to a lower switching current for current-induced magnetization switching in conventional heavy metal/ferromagnetic bilayer systems^9,15^. In addition, the switching process is reversed once the external field becomes sufficiently large to overcome the exchange bias between the CoFe and IrMn layers. A similar but opposite switching process is observed when the CoFe layer is pinned along the negative *x*-axis, for which the data are plotted in Fig. 4(b,d), similar to Fig. 4(a,c). The field effects observed in Fig. 4(c,d) are quite similar to those that occur under an applied field with the opposite polarity.

### Summary

We investigated current-induced magnetization switching with the combination of spin current due to spin-orbit interaction in the Pt layer and interlayer coupling between Co and CoFe, which is mediated by a Ta spacer layer. Because Ta has much weaker interlayer coupling strength than Ru, the thickness of a Ta spacer layer can be minimized to 0.5 nm to induce strong interlayer coupling and enable current-induced switching, even in the absence of an applied field. Such interlayer coupling plays a key role in current-induced magnetization switching in heavy metal/ferromagnetic bilayers; thus, designing and tailoring the spacer layer with Ta successfully realized SOT magnetization switching in our junction. Furthermore, the junction in this study with a Ta spacer layer shows an enhanced stability to sustain its switching characteristics against external environments. Therefore, selecting the proper material for a spacer layer with an appropriate interlayer coupling strength holds great potential for both enhanced zero-field switching and optimizing junction functionality.

## Methods

### Sample preparation

The structure was deposited on a Si/SiO_2_ substrate using an AJA magnetron sputtering system with a base pressure of ≈8 × 10^−9^ Torr. The growth rates were 0.16826 $\mathring{\rm A} /{\rm{s}}$ for Ta, 0.22478 $\mathring{\rm A} /{\rm{s}}$ for Pt, 0.13309 $\mathring{\rm A} /{\rm{s}}$ for Co, 0.22085 $\mathring{\rm A} /{\rm{s}}$ for CoFe and 0.16208 $\mathring{\rm A} /{\rm{s}}$ for IrMn at an Ar pressure of 3 mTorr. The bottom Ta layer was used to enhance the PMA of Co and improve adhesion to the substrate. The top Ta layer capped the structure for protection from oxidation. The upper Pt layer thickness of 0.4 nm is the minimum thickness to ensure the perpendicular magnetic anisotropy of the Co layer in this system. Exchange bias at the CoFe/IrMn interface was established by itself during deposition. After deposition, the multi-layers were coated with ma-N 2403 and patterned into a Hall bar of 10 × 60 μm^2^ using electron-beam lithography and ion milling. The longitudinal axis of the Hall bar was aligned along the pinned axis of CoFe. The contact pads were subsequently fabricated using UV lithography, followed by the deposition of Ta(3 nm)/Cu(150 nm) and the lift-off process.

### Measurement of magnetic properties and device performance

The exchange bias at the CoFe/IrMn interface in a full structure was evaluated using a vibrating sample magnetometer (VSM) before Hall bar patterning. The extraordinary hall measurements and current-induced switching were performed using a Keithley 6221 and 2182 A with a DC and pulsed current. The external magnetic field was applied using an electrically controlled Helmholtz coil along the perpendicular and longitudinal directions of the Hall bar. All measurements were conducted at room temperature.

### Data availability

All data generated or analysed during this study are included in this published article (and its Supplementary Information files).

## Electronic supplementary material

Supplementary information
