# Supplementary material for: Current-induced magnetic switching with spin-orbit torque in an interlayer-coupled junction with a Ta spacer layer
Source: Sci Rep. 2018 Feb 28;8:3826. doi: 10.1038/s41598-018-22122-1 (PMC5830507; doi:10.1038/s41598-018-22122-1)
Supplement: Supplementary file 1 — Supplementary information [file 41598_2018_22122_MOESM1_ESM.pdf]

<Supplementary Information>

# Current-induced magnetic switching with spin-orbit torque in an interlayer-coupled junction with a Ta spacer layer

W.-Y. Kwak,[1] J.-H. Kwon,[1] P. Grünberg,[2] S. H. Han,[3] and B. K. Cho[1, 2, *]

[1] *School of Materials Science and Engineering, Gwangju Institute of Science and Technology (GIST), Gwangju 61005, Republic of Korea*

[2] *Grünberg Center for Magnetic Nanomaterials, Gwangju Institute of Science and Technology (GIST), Gwangju 61005, Republic of Korea*

[3] *Division of Navigation Science, Mokpo National Maritime University, Mokpo, 58628, Republic of Korea*

## S1. Ta thickness dependence on the magnetic properties of the Co layer.

To determine the optimum Ta thickness, we deposited two structures: junction A, Ta(3 nm)/Pt(5 nm)/Co(0.6 nm)/Pt(0.4 nm)/Ta($t_{Ta}$ nm); and junction B, Ta(3 nm)/Pt(5 nm)/Co(0.6 nm)/Pt(0.4 nm)/Ta($t_{Ta}$ nm)/CoFe(3 nm)/IrMn(15 nm)/Ta(1 nm). The Ta thickness, $t_{Ta}$, varied according to the following values: 0.3 nm, 0.5 nm, 0.7 nm, and 1.0 nm. Anomalous Hall effect (AHE) measurements were acquired to detect magnetization in the Co layer with a current of 1 mA. For a comparison of coercivity, the Hall resistance $R_H$, which is defined by $V_H/I_{DC}$, was normalized from 0 to 1 because the AHE resistance varies depending on the amount of current flowing within the Co layer [1]. As shown in Fig. S1 (a), junction A ($t_{Ta}$ = 0.3 nm) exhibits strong perpendicular magnetic anisotropy (PMA) of the Co layer with a coercivity of ≈ 400 Oe, while junction B ($t_{Ta}$ = 0.3 nm) shows a linear $R_H$-$H$ relation. Strong interlayer coupling of the Co layer with the CoFe layer with in-plane magnetization is responsible for the loss of PMA in junction B ($t_{Ta}$ = 0.3 nm). Figures S1 (b)-(d) show $R_H$ versus $H$ for both junctions A and B with $t_{Ta}$ = 0.5 nm, 0.7 nm, and 1.0 nm. For junction A, the coercive field decreases as the Ta layer

thickness increases, while all junctions sustain the PMA of the Co layer. For junction B, the junctions with $t_{Ta}$ = 0.5 and 0.7 nm show PMA of the Co layer, while the junction with $t_{Ta}$ = 1.0 nm loses the PMA of Co. The observed Hall resistance values indicate that there is an optimum condition of the Ta layer thickness for interlayer coupling and PMA of Co within junction B. Because the interlayer coupling strength is a function of $1/t_{Ta}^2$ [2], the junction with $t_{Ta}$ = 0.3 nm removes the PMA of Co likely because of strong interlayer coupling. The thickness in the case of $t_{Ta}$ = 1.0 nm significantly diminishes the PMA of Co, regardless of interlayer coupling. While the junctions with $t_{Ta}$ = 0.5 and 0.7 nm sustain the PMA of Co, a junction with $t_{Ta}$ = 0.5 nm would provide the optimum conditions for strong interlayer coupling and PMA of Co.

**S2. Current distribution in the Pt layer as a spin current source**

The amount of current flowing in a heavy metal layer was estimated from longitudinal resistance ($R_L$) measurements of Hall bar as a function of thickness ($t_{Pt}$) of heavy metal layer [3]. For current distribution in Pt layer, we fabricated Hall bars consisting of Ta(3 nm)/Pt($t_{Pt}$ nm)/Co(0.6 nm)/Cu(2 nm)/Ta(1.0 nm) with $t_{Pt}$ = 1 nm, 2 nm, 3 nm, 4 nm, and 5 nm. Assuming that the resistance is inversely proportional to $t_{Pt}$, the linear slope in $l/R_L w$ versus $t_{Pt}$ in Fig. S2 is equal to $\rho_{pt}^{-1}$, where $w$ and $l$ are the width and length of Hall bar, respectively, and $\rho_{Pt}$ is the resistivity of Pt layer. Thus, the resistivity of Pt in the junction is estimated to be $\rho_{Pt}$ = 30.9 μΩ·cm. The sheet resistance, defined as $\rho_{Pt}/t_{Pt}$, is then 61.8 Ω for the junction in this study ($t_{Pt}$ = 5 nm). Considering a parallel resistance model, 68.5% of the total current is estimated to flow through the bottom Pt layer in the junction of Ta(3 nm)/Pt(5 nm)/Co(0.6 nm)/Pt(0.4 nm)/Ta(0.5 nm)/CoFe(3 nm)/IrMn(15 nm)/Ta(1 nm), where the longitudinal resistance is 196.8 Ω.

**S3. Harmonic measurements to estimate the effective spin Hall angle.**

Harmonic measurements were conducted to estimate the effective spin Hall angle of Pt. The stack of Ta(3 nm)/Pt(5 nm)/Co(0.6 nm)/Cu(2 nm)/Ta(1 nm) was patterned to

design Hall bar with a width of 10 μm and a length of 60 μm. Because Cu is a material with negligible spin Hall angle, Pt is considered as a spin current source for the spin orbit torque to the Co layer. With sinusoidal current with a frequency of 20 Hz along the *x*-axis Hall voltage was detected along the *y*-axis using lock-in amplifier. The external in-plane magnetic field was applied from 1000 Oe to - 1000 Oe with a step of 10 Oe along the *x*- and *y*-axes. Figure S3 shows the first harmonic voltage ($V_\omega$) (a and b) and second harmonic voltage ($V_{2\omega}$) (c and d) with a current amplitude of 12 mA. As reported in previous papers, results for the first harmonic measurement showed no distinct difference depending on the direction of the external field. For the second harmonic measurement, while the slope when the magnetic field is along the *x*-axis has the same sign regardless of Co magnetization, opposite slopes were observed with the magnetic field in the *y*-axis, as in Fig. S3 (d). Based on obtained signals in Figs. S3 (a)-(d), longitudinal and transverse effective fields can be evaluated using following equations [4].

$$\Delta H_x = -2\frac{(B_x \pm 2\xi B_y)}{1-4\xi^2} \tag{1}$$

$$\Delta H_y = -2\frac{(B_y \pm 2\xi B_x)}{1-4\xi^2} \tag{2}$$

*B* is defined as the ratio of the curvature of $V_\omega$ and the slope of $V_{2\omega}$ in V-H loops and ξ is the ratio of resistance from planar Hall effect (PHE) and anomalous Hall effect (AHE), which are described as below.

$$B_x = \frac{\partial V_{2\omega}}{\partial H_x} \Big/ \frac{\partial^2 V_\omega}{\partial {H_x}^2} \quad \text{and} \quad B_y = \frac{\partial V_{2\omega}}{\partial H_y} \Big/ \frac{\partial^2 V_\omega}{\partial {H_y}^2} \tag{3), (4}$$

$$\xi = \frac{\Delta R_{PHE}}{\Delta R_{AHE}} \tag{5}$$

The ± sign in equations (1) and (2) corresponds to magnetization alignment of the ferromagnet along the *z*-axis. In our sample, ξ of 0.31 was obtained using the first harmonic measurement in the Hall bar that is rotated in plane by 45 degrees with respect to the longitudinal axis of Hall bar [5]. As shown in Fig. S3 (e), from a linear fitting in a

plot of longitudinal effective fields in terms of current density, $\Delta H_x/J$ of 52 Oe/$10^{11}\mathrm{Am}^{-2}$ is obtained. Assuming that $\Delta H_x$ is generated by the spin Hall effect of Pt, the effective spin Hall angle can be determined using the formula of $H_x = \hbar\theta_{SH}|J|/2|e|M_S t_F$ with the effective spin Hall angle of $\theta_{SH}$, current density of $J$, saturation magnetization of $M_S$, and ferromagnet thickness of $t_F$. As a result, $\theta_{SH}$ is estimated to be ~ 0.084 with $M_S = 1013$ emu/cm$^3$ and $t_F = 0.53$ nm. The estimated $\theta_{SH}$ is within a range of previously reported values of 0.04~0.12 for Pt.

## S4. Current effects on magnetic switching in a Pt/Co/Pt junction with and without interlayer coupling

AHE resistance is plotted against the applied magnetic field (perpendicular to the Hall bar plane) in Figs. S4 (a) and (b) for junction A (Ta(3 nm)/Pt(5 nm)/Co(0.6 nm)/Pt(0.4 nm)/Ta(1 nm)) and junction B (Ta(3 nm)/Pt(5 nm)/Co(0.6 nm)/Pt(0.4 nm)/Ta(0.5 nm)/CoFe(3 nm)/IrMn(15 nm)/Ta(1 nm)), respectively. For junction A, the coercive fields decrease symmetrically to be approximately zero with both currents of $\pm$ 20 mA, regardless of the current polarity. By contrast, the coercive field is shifted to one side depending on the current flow direction for junction B, which is described in the main text. The reduction of the switching field in junction B is likely caused by thermal effects [6]. Therefore, the distinct difference in field-driven magnetization switching with a high current indicates that a visible change in switching characteristics is caused by the current-induced effective field because of interlayer coupling of Co with the CoFe layer. Figures S4 (c) and (d) illustrate the coercive field in terms of the DC amplitude. The coercive field for junction A is an almost symmetric function of the applied current along the positive and negative directions, whereas it is non-symmetric for junction B.

## S5. Axial magnetic field effects on current-induced magnetization switching in a Pt/Co/Pt junction with and without interlayer coupling

Figures S5 (a) and (b) illustrate current-induced magnetization switching for two

junctions: junction A (Ta(3 nm)/Pt(5 nm)/Co(0.6 nm)/Pt(0.4 nm)/Ta(1 nm)) and junction B (Ta(3 nm)/Pt(5 nm)/Co(0.6 nm)/Pt(0.4 nm)/Ta(0.5 nm)/CoFe(3 nm)/IrMn(15 nm)/Ta(1 nm)), respectively. A pulsed current with a 1 ms duration was injected along the $x$-axis of the Hall bar, and voltage was measured at the centre of each pulse. A constant external field along the $x$-axis was applied over the range – 600 Oe ≤ $H$ ≤ + 600 Oe with a step of 100 Oe. For junction A, magnetic switching of Co with PMA does not occur when $H$ = 0 Oe; meanwhile, the current-induced magnetic switching starts to occur when a magnetic field is applied, as shown in Fig. S5 (a). The switching sequence is clockwise with a positive field and counter-clockwise with a negative field. This pattern indicates that the applied field plays a role in symmetry breaking for the current-induced magnetic switching of Co. However, the current-induced magnetic switching occurs even when $H$ = 0 Oe for junction B. In addition, the switching sequence is maintained as clockwise with the applied fields except when $H$ = – 600 Oe, wherein the switching sequence becomes counter-clockwise. This finding indicates that interlayer coupling acts solely as a symmetry breaker, similar to the positive field in Fig. S5 (a) and that the coupling effect is cancelled by the applied field of $H$ = – 600 Oe. Figures S5 (c) and (d) show the critical current density for magnetic switching in terms of an applied magnetic field for junctions A and B, respectively. For junction A, the critical current decreases as the applied field increases regardless of the directionality of current, which is consistent with the behaviour in a heavy metal/ferromagnet bilayer system [6,7]. For junction B, the field effect is the same as that for junction A as long as the field is less than the interlayer coupling strength if it weakens the coupling or enhances the coupling strength.

## References

[1] Kim, J. *et al.*, Layer thickness dependence of the current-induced effective field vector in Ta|CoFeB|MgO. *Nat. Mater.* **12**, 240-245 (2013).

[2] Parkin, S. S. P., Systematic variation of the strength and oscillation period of indirect magnetic exchange coupling through the 3d, 4d, and 5d transition metals. *Phys. Rev. Lett.* **67**, 3598-3601 (1991).

[3] Ramaswamy, R., Qiu, X., Dutta, T., Pollard, S. D. & Yang, H. Hf thickness dependence of spin-orbit torques in Hf/CoFeB/MgO heterostructures. *Appl. Phys. Lett.* **108**, 202406 (2016).

[4] Hayashi, M., Kim, J., Yamanouchi, M. & Ohno, H. Quantitative characterization of the spin-orbit torque using harmonic Hall voltage measurements. *Phys. Rev. B* **89,** 144425 (2014).

[5] Woo, S., Mann, M., Tan, A. J., Caretta, L. & Beach. G. S. D. Enhanced spin-orbit torques in Pt/Co/Ta heterostructures. *Appl. Phys. Lett.* **105**, 212404 (2014).

[6] Liu, L., Lee, O. J., Gudmundsen, T. J., Ralph, D. C. & Buhrman, R. A. Current-induced switching of perpendicularly magnetized magnetic layers using spin torque from the spin Hall effect. *Phys. Rev. Lett.* **109**, 096602 (2012).

[7] Hin Sim, C., Cheng Huang, J., Tran, M. & Eason, K. Asymmetry in effective fields of spin-orbit torques in Pt/Co/Pt stacks. *Appl. Phys. Lett.* **104**, 012408 (2014).

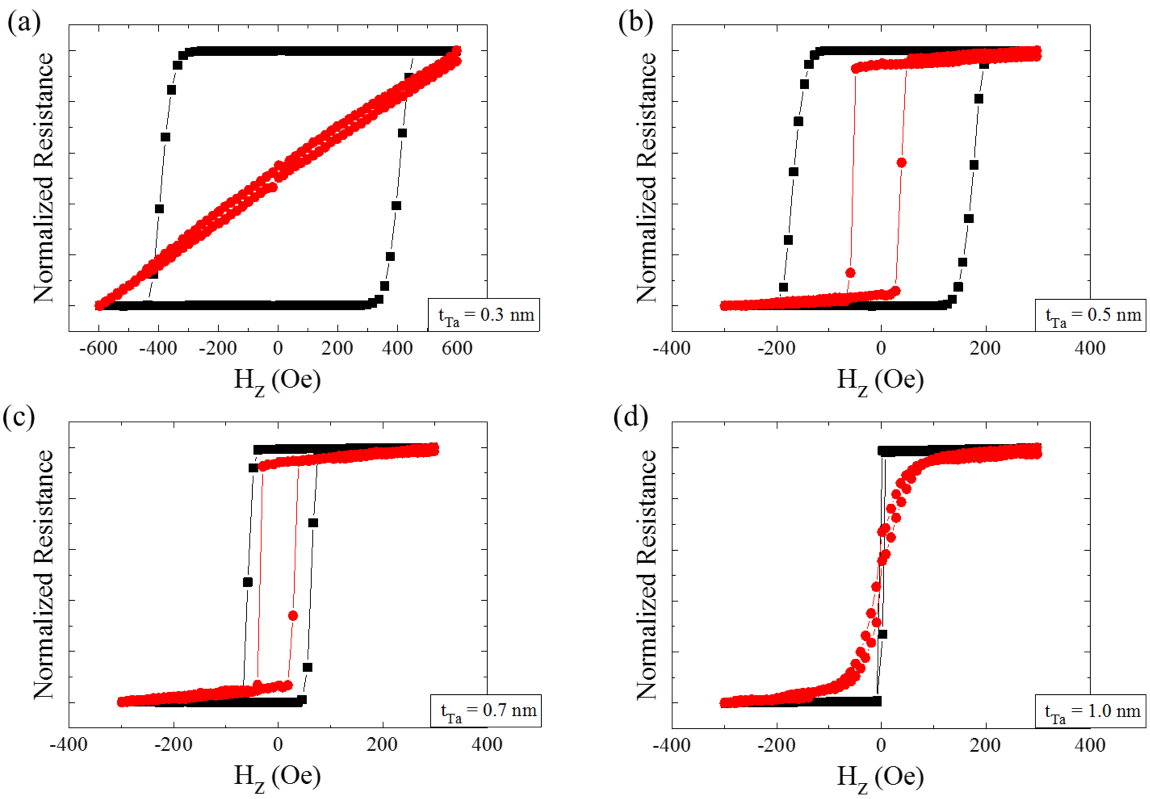


**Figure S1. Ta thickness effect on the magnetic properties of the Co layer. (a)-(d)** Normalized Hall resistance with various Ta thicknesses ($t_{Ta}$) for junction A (black square); Ta(3 nm)/Pt(5 nm)/Co(0.6 nm)/Pt(0.4 nm)/Ta($t_{Ta}$ nm)) and junction B (red circle); Ta(3 nm)/Pt(5 nm)/Co(0.6 nm)/Pt(0.4 nm)/Ta($t_{Ta}$ nm)/CoFe(3 nm)/IrMn(15 nm)/Ta(1 nm)).

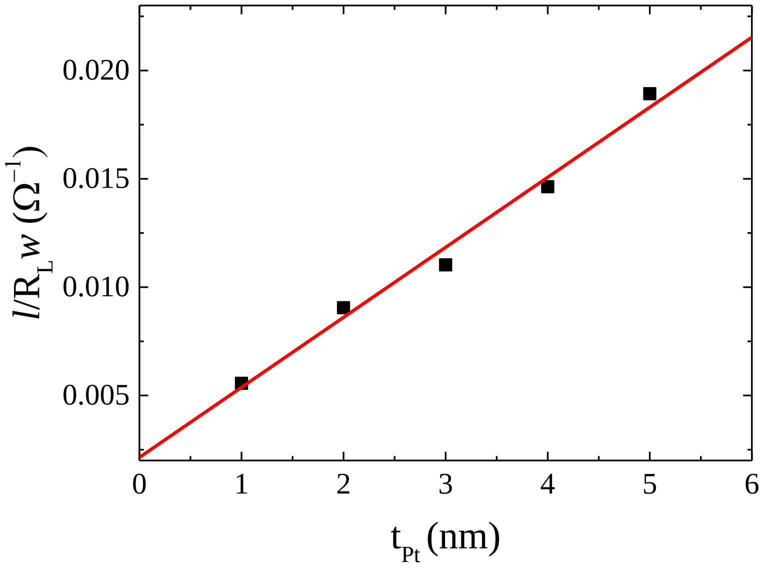


**Figure S2. Current distribution in the Pt layer.** Length of the Hall bar divided by the longitudinal resistance and the width of bars as a function of Pt thickness ($t_{Pt}$) for a junction composed of Ta(3 nm)/Pt($t_{Pt}$ nm)/Co(0.6 nm)/Cu(2 nm)/Ta(1 nm).

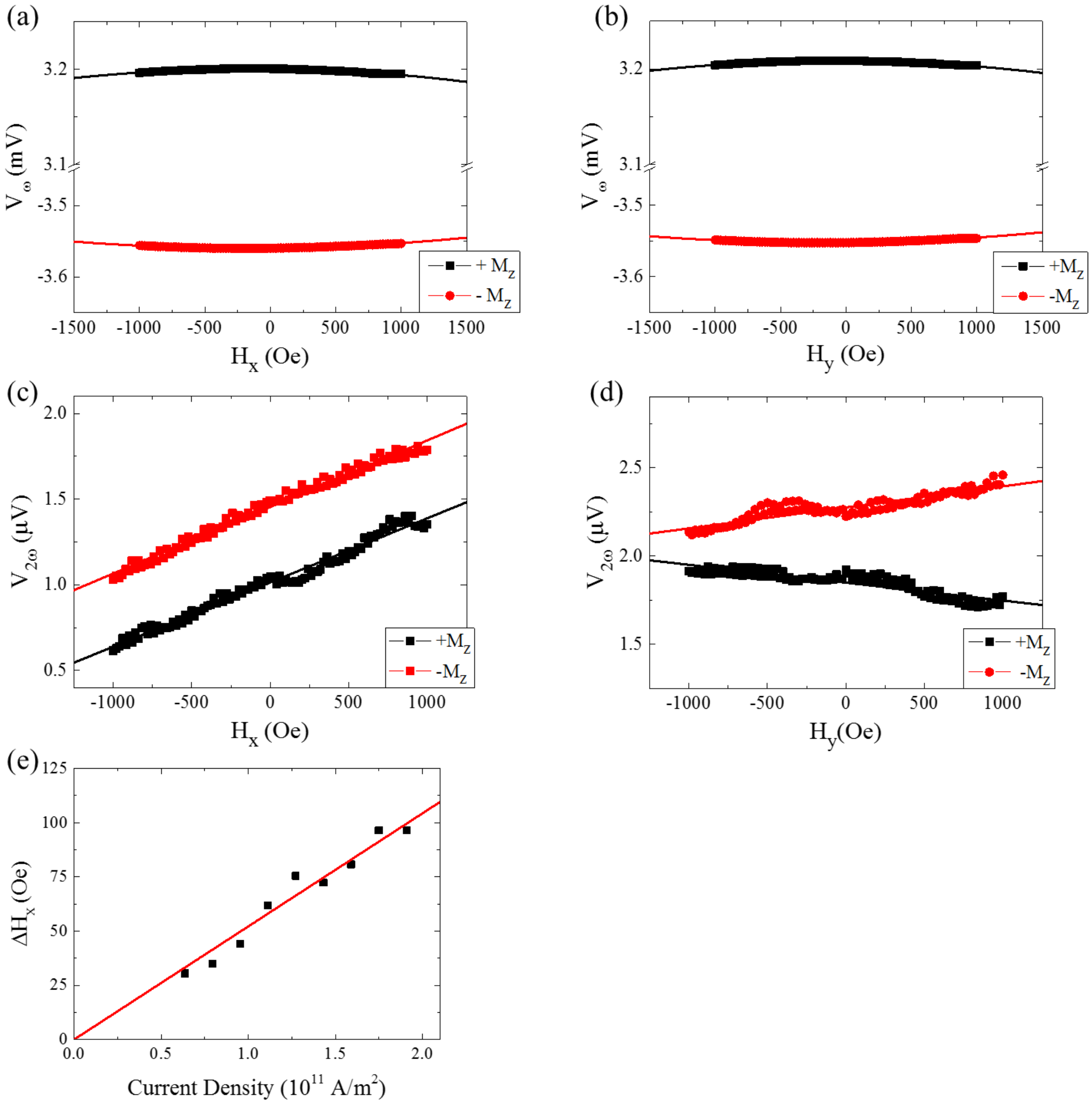


**Figure S3. Harmonic measurement for the estimation of effective spin Hall angle of Pt.** (**a**),(**b**) First harmonic voltage as a function of the external magnetic field along *x*- and *y*-axes. (**c**),(**d**) Second harmonic voltage as a function of the external magnetic field along *x*- and *y*-axes. (**e**) Plot of longitudinal effective fields from the spin-orbit torque in terms of current density in Pt.

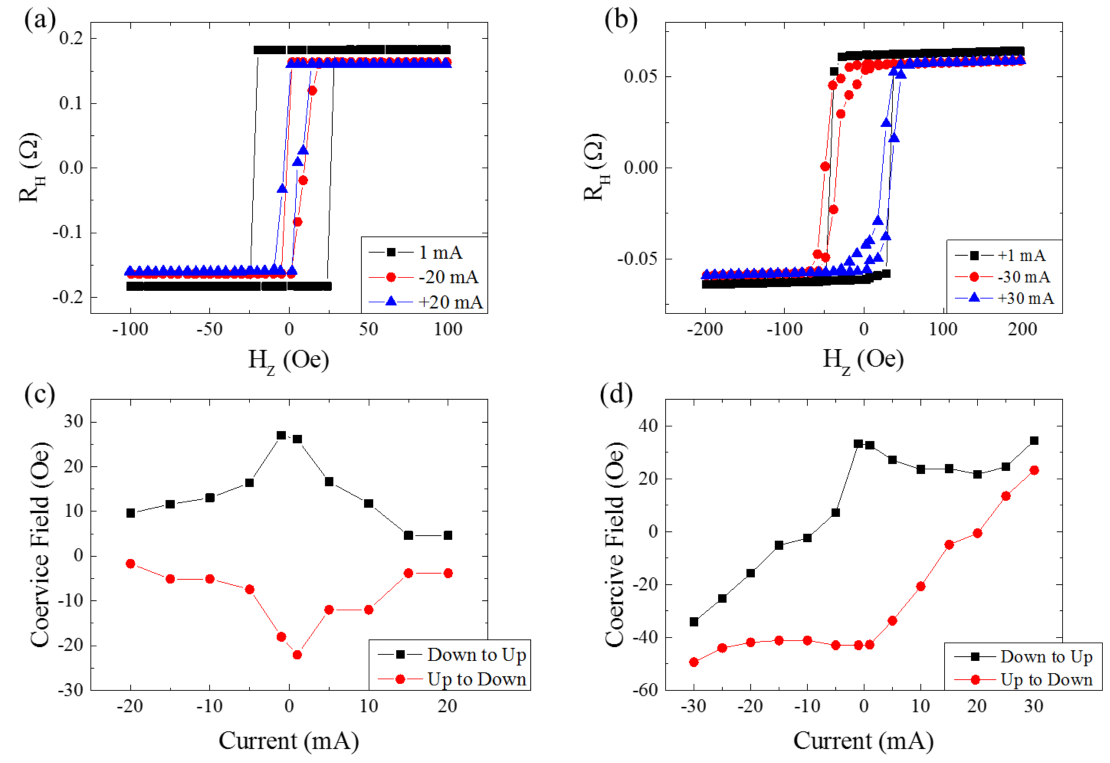


**Figure S4. Current effect on magnetic switching in a Pt/Co/Pt structure.** (**a**),(**b**) Field-driven magnetization switching behaviour with various currents for junction A (Pt(5 nm)/Co(0.6 nm)/Pt(0.4 nm)) and junction B (Pt(5 nm)/Co(0.6 nm)/Pt(0.4 nm)/Ta(0.5 nm)/CoFe(3 nm)/IrMn(15 nm)), respectively. (**c**),(**d**) Plots of the coercive field with respect to the applied current in junctions A and B, respectively.

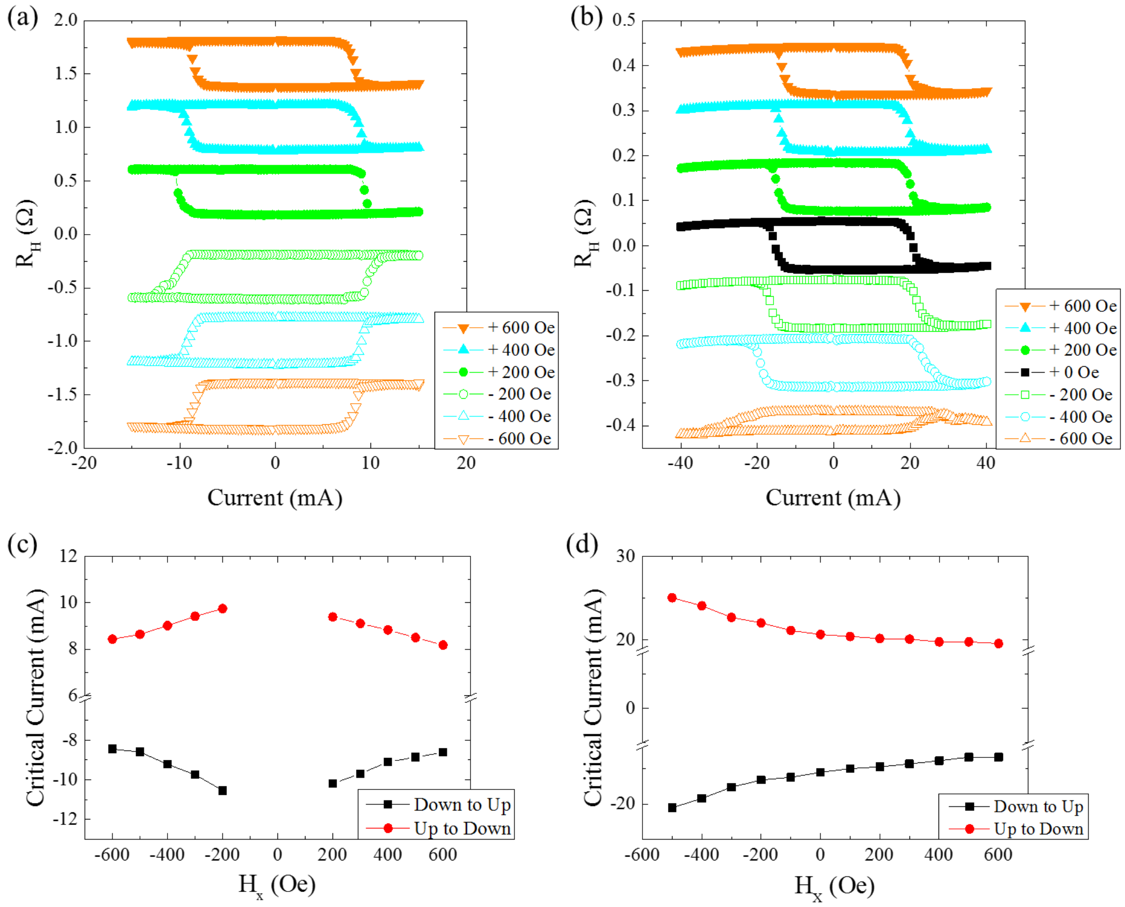


**Figure S5. Current-induced magnetization switching of a Pt/Co/Pt structure under axial magnetic fields.** **(a)**,**(b)** Current-induced magnetization switching for junction A (Pt(5 nm)/Co(0.6 nm)/Pt(0.4 nm)) and junction B (Pt(5 nm)/Co(0.6 nm)/Pt(0.4 nm)/Ta(0.5 nm)/CoFe(3 nm)/IrMn(15 nm)), respectively. **(c)**,**(d)** Plots of the critical current with respect to the axial magnetic field for junctions A and B, respectively.
